# Right ventricle late gadolinium enhancement in cardiac sarcoidosis: a case series

**DOI:** 10.1186/1532-429X-13-S1-P300

**Published:** 2011-02-02

**Authors:** Carmen P Lydell, Iacopo Carbone, Naeem Merchant

**Affiliations:** 1 University of Calgary, Calgary, AB, Canada

## Objective

To determine the prevalence of left and right ventricle late gadolinium enhancement in a series of patients with sarcoidosis and suspected cardiac involvement referred for cardiac MRI.

## Background

Sarcoidosis is a multisystem disease characterized by infiltration of tissues by non-caseating granulomas and cardiac disease associated with arrhythmias, conduction disturbances, heart failure and death. Previous case reports have described late gadolinium enhancement (LGE) in the right ventricle (RV), however, this has been interpreted to be a rare feature of cardiac sarcoidosis. We identified a cases series of patients with sarcoidosis and evaluated the presence of LGE in the RV and LV.

## Methods

Patients referred for cardiac MRI with any notation of “sarcoidosis” between 2007-2010 were identified. Charts were subsequently reviewed to confirm a clinical diagnosis of sarcoidosis based on clinical history and pathology results and characteristic chest CT findings. A total of 47 patients with possible sarcoidosis were identified. Seventeen patients had confirmatory diagnosis of sarcoidosis based on tissue diagnosis or a strong clinical suspicion with characteristic CT chest findings and were included in this case-series.

## Results

Of the 17 patients, 12 (71%) showed LGE in the LV myocardium and 7 (41%) showed LGE in the RV myocardium. All patients with RV involvement also had LV involvement. Five patients showed no LGE in either the LV or RV. The pattern of RV enhancement was typically transmural.

## Conclusion

RV involvement in sarcoidosis may be more common than previously appreciated and appears to and coexists with similar findings in the LV. Larger, more rigorous studies are warranted to assess the prevalence of this feature and it’s sensitivity for the diagnosis of cardiac sarcoidosis.

